# Bilateral endogenous fungal endophthalmitis

**DOI:** 10.1007/s10792-013-9783-x

**Published:** 2013-05-03

**Authors:** Wilczynski Michal, Wilczynska Olena, Omulecki Wojciech

**Affiliations:** Department of Ophthalmology, University Barlicki Hospital No. 1, Medical University of Lodz, ul.Kopcinskiego 22, 90-153 Lodz, Poland

**Keywords:** Fungal endogenous endophthalmitis, Pars plana vitrectomy, Postoperative complications, *Candida albicans*

## Abstract

Endogenous endophthalmitis is a rare and severe intraocular infection which can be vision-threatening. We describe a case of bilateral fungal endogenous endophthalmitis in a 64-year-old male which was successfully treated with systemic administration of fluconazole followed by pars plana vitrectomy with an intravitreous injection of amphotericin B.

## Introduction

Endogenous endophthalmitis is a severe infrequent disease which can lead to loss of vision.

We describe a case of bilateral fungal endogenous endophthalmitis in a 64-year-old male which was successfully treated with intravenous and oral fluconazole followed by pars plana vitrectomy and an intravitreous injection of amphotericin B.

## Case report

A 64-year-old Caucasian male was referred to the Department of Ophthalmology, Medical University of Lodz (Poland) to undergo planned consultation for decreased vision.

The patient had been treated in the Department of General and Transplantation Surgery where he underwent a pancreatoduodenectomy (by Whipple) as treatment for carcinoma of the distal part of the bile duct and a right-sided hemicolectomy as a treatment of complications which developed after the first surgery.

After these operations, the patient received long-standing intravenous feeding and intensive general antibiotic therapy, which was based on bacterial cultures and antibiogram. The patient’s general condition was considered serious at that time. Three weeks after the first operation, the patient noticed bilateral deterioration of vision with ‘spider net’ floaters and ocular pain.

**Fig. 1:**
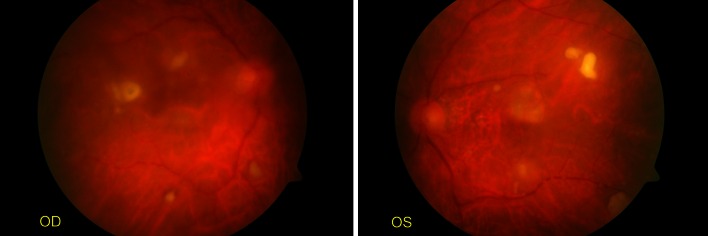
Early manifestation of fungal endophthalmitis. Multifocal retinitis is visible, as well as inflammatory exudates in the vitreous (*OD*, the right eye; *OS*, the left eye)

During the first ophthalmic examination his best-corrected visual acuity (BCVA) was 0.02 in both eyes, and intraocular pressure (IOP) was 12 mm Hg. Slit-lamp examination revealed bilateral ciliary engorgement and posterior synechiae. In the eye fundus examination, convex, whitish, well-defined inflammatory lesions of 0.5 mm diameter were present on the retina (multifocal retinitis) and inflammatory exudates were present in the vitreous. Juxtafoveal lesions were seen in both eyes—in the right eye three of these lesions were present on the edge of the macula and in the left eye one lesion was present in the upper part of the macula (Fig. 1). Bilateral endogenous endophthalmitis was diagnosed. As the clinical appearance suggested fungal etiology, the patient was offered an intravitreous injection of amphotericin B but did not agree to undergo this treatment. At this point the patient also refused to undergo pars plana vitrectomy.

Intravenous fluconazole 200 mg/day was administered, as well as topical treatment with 1 % tropicamide eyedrops.

After treatment, the symptoms of anterior uveitis resolved within 2 days. Fungal culture from a blood sample was performed three times with negative results each time.

During the following week a further decrease of visual acuity was observed (BCVA of 0.01 in both eyes). In the vitreous the exudates were thicker and there were numerous ‘cotton ball’ colonies present. There were numerous small round whitish spots located along the larger retinal vessels.

**Fig. 2:**
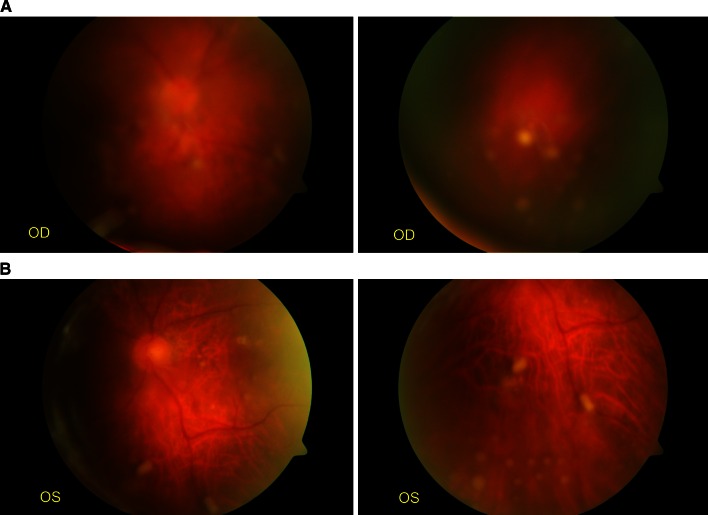
**a, b** Bilateral endogenous fungal endophthalmitis one week later (*OD*, the right eye; *OS*, the left eye)

**Fig. 3:**
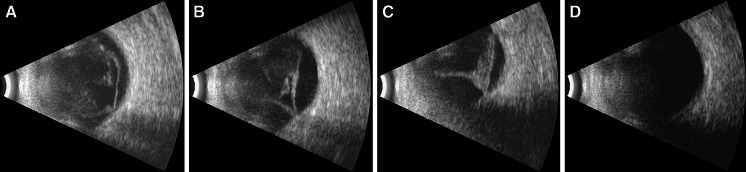
Consecutive ultrasound scans, showing gradual thickening of vitreous exudate and posterior vitreous detachment (**a**–**c**) and clear vitreous chamber after pars plana vitrectomy (**d**)

A few days later the patient’s general condition and ophthalmic condition both improved. Retinal lesions started to decrease in size and became flatter and paler. At the same time visual acuity improved to 0.1 in both eyes, and a week later it improved to 0.2. Nevertheless, the vitreous exudate became thicker and more condensed (Figs. 2, 3). In the next few days, posterior vitreous detachment developed and visual acuity decreased to 0.02 in both eyes.

The patient was discharged from the Department of General Surgery and was admitted to the Department of Ophthalmology where he underwent pars plana vitrectomy with an intravitreous injection of amphotericin B in both eyes. This treatment resulted in a marked increase in visual acuity (BCVA in the right and left eye was 0.5 and 0.3, respectively). Vitreous tap was taken during vitrectomy and samples of vitreous fluid were sent for cultures (bacteriologic and mycological) and direct specimens were made. The culture results were negative; however, direct specimens revealed the presence of *Candida* sp. The patient received fluconazole intravenously and orally for a total of 3 months.

After the active inflammatory reaction was controlled pharmacologically, retinal and choroidal scars were present in both eyes. In the left eye one of these scars was located in the upper part of the macula.

One year after the initial diagnosis of endophthalmitis, the patient’s visual acuity amounted to BCVA of 0.4 in the right eye and 0.1 in the left eye. Mild nuclear cataracts have recently been found in both eyes.

## Discussion

Endogenous endophthalmitis is a rare and severe intraocular infection which can be vision-threatening and results in total loss of vision [1]. It is usually a result of hematogenous dissemination of pathogens from another site of coexisting infection.

Fungi are the most commonly encountered microorganisms that cause endogenous endophthalmitis. *C. albicans* is the most frequent pathogen, accounting for approximately 75–80 % of fungal infections [1, 2]. *Candida* fungi are commensal organisms, which can cause infections in the immunosuppressed [3].

There are many risk factors connected to endogenous endophthalmitis, the most important being intravenous antibiotic treatment, major surgery, intravenous catheters, intravenous infusions, steroid treatment, therapy with immunosuppresive agents and intravenous drug abuse [1]. Fungal dissemination is particularly frequent in diabetic patients, neonates, and burn patients [3]. It may lead to endocarditis, meningitis, arthritis, choroiditis, retinitis or endophthalmitis [3].

Patients after gastrointestinal tract surgery tend to have compromised circulation in the involved tissues and often undergo intravenous broad-spectrum antibiotic treatment afterwards, which may be a predisposing factor to *Candida* infiltration [1]. Our patient had undergone major gastroinestinal tract surgery, which led to prolonged intravenous broad-spectrum antibiotic treatment combined with prolonged intravenous feeding, which in turn caused endogenous fungemia.

The main result of ocular candidiasis is a chorioretinitis; however, the infection can spread to the optic nerve, vitreous, and ciliary body and cause endophthalmitis or panophthalmitis. It is estimated that in approximately 67 % of patients the disease is bilateral and in about 80 % of patients the lesions are multifocal [2, 4].

Patients may experience changes in visual acuity, scotomas, floaters, photophobia or pain. In most patients, macular or vitreous lesions are present, which are usually described as round and whitish, small, focal, perivascular, chorioretinal lesions with surrounding vitreous inflammation (vitritis). They are also referred to as white fluffy exudates with well-circumscribed borders. Vascular sheathing, Roth’s spots, and exudative retinal detachment may also be present. In addition, anterior segment inflammation (anterior uveitis) may develop [1, 2].

Usually, the diagnosis is based on history and clinical findings; however, it should be confirmed by isolating the microorganism from infected tissues (blood, aqueous fluid or vitreous) [2].

The prognosis for endogenous endophthalmitis is usually poor, which results from the fact that patients are frequently immunocompromised, the microorganism is virulent and diagnosis is often delayed [1, 2].

Endogenous endophthalmitis responds better to intravenous antibiotics than exogenous endophthalmitis. It is important to make a diagnosis as early as possible and to start appropriate treatment immediately. It is therefore crucial to identify the causative agent correctly. Blood or aqueous humor cultures and determining the susceptibility of the microorganisms to various antifungal drugs help to choose the most effective treatment [1].

In the described case, fungal culture from a blood sample was performed three times and was negative on each occasion, so the initial diagnosis was based on the clinical picture. Later in the course of the disease direct specimens from the vitreous tap confirmed the presence of *Candida* sp. Another useful diagnostic option for detecting the DNA of *Candida* species is broad-range real-time polymerase chain reaction; however, this method was unavailable to us [5].

In our patient, juxtafoveal lesions were seen in both eyes as well as vitritis, which implies poor final visual prognosis and is an indication for intravitreous injection or vitrectomy. As the patient at first refused an intravitreous injection of amphotericin B and pars plana vitrectomy, only intravenous fluconazole was started. Pars plana vitrectomy was finally performed about a week after the patient’s initial visit, which might have influenced the final visual acuity.

In ocular candidiasis it is advised that treatment should be commenced as quickly as possible; however, there are studies showing that, even when the treatment is commenced on time, final visual acuity is poor [6]. This is often a result of macular photoreceptor damage, which was also the case in our patient, who was later found to have bilateral chorioretinal scars in the macular area.

In a study by Sallam et al. [7], 55 % of eyes with *Candida* endophthalmitis had visual loss (defined as visual acuity of <20/40) and 32 % had severe visual loss (defined as visual acuity of <20/200). Authors concluded that *Candida* endophthalmitis was associated with a high rate of visual loss and they also found that early vitrectomy reduced the risk of retinal detachment significantly. Poor presenting visual acuity and centrally located fungal lesions were found to be the main factors associated with poor visual outcome [7].

It is thought that early systemic treatment should be used, either with imidazoles or amphotericin B [1, 8, 9]. In cases of progressive vitritis, vitrectomy should be performed to remove the microorganisms and intravitreal amphotericine B should be used in conjunction with vitrectomy [2, 8, 9].

Our patient agreed to undergo pars plana vitrectomy only when visual acuity dropped again after it initially increased, as a result of the introduction of intravenous fluconazole.

In the literature it is stressed that oral fluconazole may be used as an alternative treatment to intravitreal amphotericine B, and may be used after vitrectomy for a prolonged period of time, especially in cases of *Candida* endophthalmitis which are resistant to amphotericin B [1, 8–10].

In our case, when the patient refused vitrectomy, we decided to introduce systemic fluconazole, which was continued for approximately 3 months after vitrectomy with intravitreal amphotericine B administration. In the literature, there are reports stating that bilateral endogenous *Candida* endophthalmitis can be successfully treated with pars plana vitrectomy and intravenous fluconazole alone [11]. Some authors advocate also using systemic corticosteroids in addition to amphotericin B; however, it is advised to start steroids no sooner than 48 h after antifungal therapy [2, 10].

The present case report demonstrates that intravenous and oral fluconazole followed by pars plana vitrectomy with an intravitreous injection of amphotericin B, is an effective treatment for endogenous fungal endophthalmitis. Direct specimens of the vitreous tap material should be made, as they allow identification of the causative pathogen even in cases of negative culture results.
